# Effects of Exogenous Phosphorus and Hydrogen Peroxide on Wheat Root Architecture

**DOI:** 10.3390/plants15020253

**Published:** 2026-01-13

**Authors:** Lei Chen, Lei Zhou, Yuwei Zhang, Hong Wang

**Affiliations:** 1 Heilongjiang Academy of Black Soil Conservation and Utilization, No. 368, Xuefu Road, Harbin 150086, China; chengz5251@163.com; 2 Heilongjiang Academy of Sciences, No. 204, Zhongshan Road, Harbin 150001, China; boshihou5@163.com; 3 Institute of Agricultural Resources and Regional Planning, Chinese Academy of Agricultural Sciences, No. 12, Zhongguancun South Road, Beijing 100081, China; hljhty@126.com

**Keywords:** hydrogen peroxide, phosphorus, root, winter wheat, oxidative enzyme, fluorescence reaction

## Abstract

Plant root growth and architectural modifications are well-documented responses to phosphorous (P) starvation. The spatiotemporal dynamics of hydrogen peroxide (H_2_O_2_) in mediating root development under P deficiency, especially in cereal crops like wheat, remain insufficiently understood. A nutrient solution experiment was conducted to grow two varieties of wheat, including SM15 and HG35, with the treatments of 0.005 and 0.25 mmol/L P supply. Exogenous H_2_O_2_ and its scavenger ascorbic acid (AsA), and a NADPH oxidase inhibitor diphenylene iodonium (DPI) were added. The distribution of reactive oxygen species (ROS) in roots were detected by chemical staining and fluorescent probe technology. Low P supply did not change the root dry weight and total root length, while it decreased the lateral root density. The increase in the primary root and lateral root growth in P-starved wheat coincided with more ROS in the cell wall of the elongation zone. ROS production and oxidative enzyme activity of P-starved roots increased significantly. Low H_2_O_2_ induced the formation of lateral roots and significantly increased lateral root density under low P conditions. High H_2_O_2_ significantly reduced lateral root density but stimulated the nodal root formation. Exogenous AsA or DPI addition reversed the promotion of root growth imposed under the low P treatment or H_2_O_2_ addition. Furthermore, exogenous H_2_O_2_ treatment reduced the inhibitory effect of the DPI treatment on nodal root formation. It is suggested that the involvement of ROS in the regulation of wheat root system architecture under low P supply.

## 1. Introduction

Phosphorus (P) is a nutrient essential for plant growth and development. Its availability in soil is often critically low due to strong sorption onto mineral and organic surfaces and slow diffusion rates, making P deficiency a primary constraint on crop productivity [1,2]. To adapt, plants employ sophisticated signaling networks that orchestrate a suite of morphological and physiological responses. A cornerstone of adaptation is the remodeling of root system architecture (RSA). Under phosphate (Pi) deficiency, a typical strategy involves the inhibition of primary root elongation coupled with the promotion of lateral root formation and root hair elongation, thereby expanding the root surface area in topsoil layers where Pi may be relatively more accessible [3,4]. Under low-P conditions, the efficiency of P acquisition is governed more by root morphological traits (e.g., root-hair density, root elongation) than by the kinetic parameters of phosphate transporters [1].

Concomitant with these developmental changes, P deficiency perturbs the cellular metabolism [4], often leading to the accumulation of ROS-like superoxide (O_2_^•−^), H_2_O_2_ [5,6,7]. While excessive ROS can cause oxidative damage, they also function as pivotal signaling molecules that modulate growth and stress responses [5]. Plants maintain redox homeostasis through a sophisticated scavenging system involving enzymes such as superoxide dismutase (SOD), catalase (CAT), and peroxidase (POD), as well as antioxidants like ascorbic acid (AsA) and glutathione (GSH) [5,6,8].

Accumulating evidence implicates ROS as key regulators linking P status to RSA modification. In *Arabidopsis thaliana*, P deficiency alters the spatial pattern of ROS in root tips, shifting accumulation from the elongation zone to the apical meristem, which correlates with inhibited primary root growth and stimulated lateral root formation [9,10]. Mechanistically, ROS accumulation can increase callose deposition in cell walls and plasmodesmata (PD), potentially hindering the intercellular trafficking of key developmental regulators like the SHORT-ROOT (SHR) protein, thereby affecting root meristem maintenance [11,12]. Supporting this, proteomic studies indicate that specific class III peroxidases are regulated in P-deficient root tips, suggesting a direct link between ROS metabolism, POD activity, and cell wall remodeling during adaptive root growth [13]. Observations in bean plants further corroborate this connection, showing that P starvation elevates H_2_O_2_ levels and the activities of antioxidant enzymes like CAT and POD [14,15].

Despite these advances, a detailed understanding of the spatiotemporal dynamics of ROS and the associated enzymatic activities in mediating root development under P deficiency, particularly in cereal crops like wheat, remains incomplete. Moreover, while the dual-role of ROS in root development has been documented in model species, the concentration-dependent effects of H_2_O_2_ and its interaction with P availability in wheat are not well-defined. This study therefore aimed to accomplish the following: (1) characterize the changes in RSA and ROS distribution in two wheat cultivars under low- and sufficient-P conditions; (2) examine the activities of key antioxidant enzymes (SOD, POD, CAT); and (3) elucidate the effects of exogenous H_2_O_2_, its scavenger AsA, and the NADPH oxidase inhibitor DPI on root architectural responses. By integrating morphological, histochemical, and biochemical analyses, we provide new insights into how ROS signaling modulates wheat RSA under P limitation, highlighting cultivar-specific adaptations and the dose-dependent nature of H_2_O_2_ action.

## 2. Results

### 2.1. Plant Biomass

In two wheat varieties (SM15 and HG35), seedlings grown in low-P medium exhibited typical P-deficiency symptoms 15 days after treatment (DAT), including reduced leaf size and chlorotic leaf tips. Under low-P conditions, root biomass and root/shoot ratio were significantly higher than those under normal-P supply, whereas shoot biomass decreased compared to the control group (Table 1).

Exogenous application of 10.0 mmol/L H_2_O_2_ significantly inhibited root growth and reduced both shoot and root biomass across all experimental groups, independent of wheat variety or P supply level. Lower H_2_O_2_ concentrations (1.0 mmol/L) significantly decreased shoot and root biomass in low-P medium but had no observable effect on root biomass in normal-P medium (Table 1).

When applied without H_2_O_2_ pretreatment, both AsA (2.5 mmol/L) and DPI (5.0 μmol/L) significantly reduced shoot and root dry weights (Table 1). Notably, 2.5 mmol/L AsA failed to counteract the biomass inhibition caused by 1.0 mmol/L H_2_O_2_, yet effectively mitigated the root biomass suppression induced by 10.0 mmol/L H_2_O_2_. Conversely, co-treatment with 1.0 mmol/L H_2_O_2_ reversed DPI-induced reductions in shoot and root dry weights under both P supply regimes.

AsA and DPI significantly decreased root/shoot ratios under low-P conditions (with or without 1.0 mmol/L H_2_O_2_) but showed no effect under sufficient-P conditions (Table 1). The suppressive effect of 10.0 mmol/L H_2_O_2_ on the root-shoot ratio was partially alleviated by 2.5 mmol/L AsA, with the combined treatment yielding significantly higher ratios than H_2_O_2_ alone. In low-P medium, 1.0 mmol/L H_2_O_2_ + 5.0 μmol/L DPI co-treatment increased the root/shoot ratio compared to DPI alone, suggesting H_2_O_2_’s protective role against DPI-induced alterations. For HG35 under P sufficiency, 1.0 mmol/L H_2_O_2_ not only counteracted DPI’s effect on the root-shoot ratio but enhanced this parameter relative to controls.

### 2.2. P Concentration in Shoots

Compared with adequate-P supply, low-P conditions significantly reduced shoot P concentration in both wheat varieties. Notably, under sufficient-P conditions, HG35 shoots exhibited a lower P concentration than SM15 shoots (Table 1).

H_2_O_2_ treatment under adequate-P supply significantly decreased shoot P concentration. In contrast, 1.0 mmol/L and 10.0 mmol/L H_2_O_2_ increased the P concentration in shoots of SM15 by 9.3% and 18.7% compared with no H_2_O_2_ treatment under low-P conditions, respectively.

While 2.5 mmol/L AsA exerted no significant influence on shoot P concentration under low-P conditions, it markedly decreased P levels (*p* < 0.05) in shoots under optimal-P supply. Notably, co-application of 10.0 mmol/L H_2_O_2_ attenuated AsA’s inhibitory effect on P concentration in normal-P environments. In sufficient-P medium, 5.0 μmol/L DPI treatment significantly reduced shoot P concentration by 36.2~55.2% (vs control), and H_2_O_2_ supplementation failed to counteract this DPI-induced suppression across all P regimens in both wheat varieties (Table 1).

### 2.3. Root Traits

Compared with normal-P supply, low-P conditions significantly enhanced primary root length, inducing increases of 17.8% in SM15 and 18.5% in HG35 (Table 2). Lateral roots exhibited distinct morphological responses: average lateral root length increased by 34.4% (SM15) and 14.7% (HG35) under P deficiency, whereas lateral root number and density decreased by 27.5% (SM15) and 25% (HG35) compared to normal-P conditions. Contrastingly, optimal-P supply elevated root length density by 9.0% (SM15) and 12.9% (HG35) relative to low-P treatments, demonstrating P availability’s dual regulatory effects on root architecture.

All chemical treatments (10.0 mmol/L H_2_O_2_, 2.5 mmol/L AsA, and 5.0 μmol/L DPI) consistently suppressed root system development across P regimes, significantly reducing total root length (TRL), primary root length (PRL), nodal root length (NRL) and lateral root length (LRL) (vs. untreated controls). Notably, AsA supplementation mitigated the strong inhibitory effect of 10.0 mmol/L H_2_O_2_ on root elongation, with 1.0 mmol/L H_2_O_2_ + AsA co-treatment under P sufficiency partially reversing AsA-induced suppression (TRL: +2.4~4.9% vs. AsA alone; Table 2). For SM15 under 0.25 mmol/L P supply, H_2_O_2_-DPI interactions exhibited concentration-dependent effects: 10.0 mmol/L H_2_O_2_ + DPI significantly alleviated DPI-induced PRL (+24.0%) and TRL (+39.9%) reductions (Table 2).

Compared to untreated controls, 10.0 mmol/L H_2_O_2_ treatment markedly enhanced nodal root proliferation, while 2.5 mmol/L AsA exerted a synergistic stimulatory effect, contrasting with 5.0 μmol/L DPI’s significant suppression of this trait. Notably, combinatorial treatments revealed concentration-dependent modulation: both 1.0 mmol/L H_2_O_2_ + 2.5 mmol/L AsA and 1.0 mmol/L H_2_O_2_ + 5.0 μmol/L DPI significantly increased nodal root numbers (by 50~80% and 20~60%, respectively) compared to 1.0 mmol/L H_2_O_2_ alone. Critical regulation patterns emerged: 2.5 mmol/L AsA and 5.0 μmol/L DPI substantially attenuated the nodal root-promoting effects of both low (1.0 mmol/L) and high (10.0 mmol/L) H_2_O_2_ concentrations, demonstrating bidirectional control of ROS homeostasis on root architecture (Table 2).

The 1.0 mmol/L H_2_O_2_ treatment significantly enhanced lateral root density (LRD) in both wheat varieties (LRD: +30.1~65.8% vs. control; Table 2), whereas high-concentration H_2_O_2_ (10.0 mmol/L) exhibited a P-dependent modulation: no significant effect under 0.005 mmol/L P but marked suppression (LRD: −22.1~23.9%) under 0.25 mmol/L P. Under low-P conditions, the lateral root density increased by 65.8% in SM15 and 50.0% in HG35. In contrast, under adequate-P conditions, the increases were 44.9% and 30.1% for SM15 and HG35, respectively. Both 2.5 mmol/L AsA and 5.0 μmol/L DPI treatments consistently reduced LRD (*p* < 0.05) compared to untreated controls. Notably, AsA selectively neutralized 1.0 mmol/L H_2_O_2_’s promotive effect (LRD: −54.9~63.9% vs. H_2_O_2_ alone) while failing to counteract 10.0 mmol/L H_2_O_2_’s suppression. Conversely, H_2_O_2_-DPI co-treatments demonstrated concentration-specific rescue effects: 1.0/10.0 mmol/L H_2_O_2_ attenuated DPI-induced LRD inhibition (Table 2), suggesting H_2_O_2_’s dual role in ROS-mediated wheat root morphogenesis.

### 2.4. ROS Distribution in the Roots

**Figure 1:**
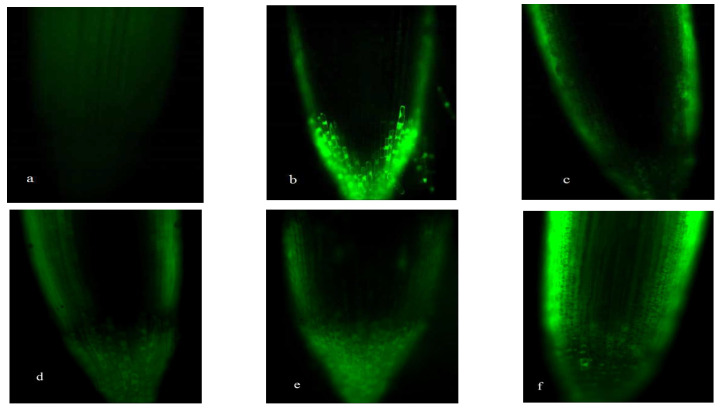
Hydrogen peroxide (H_2_O_2_) distribution in the root tips of SM15 wheat seedlings grown on a medium of two Pi concentrations and sampled at different ages. The treatment (+P): (**a**) 5 DAT, (**b**) 10 DAT, and (**c**) 15 DAT. The treatment (−P): (**d**) 5 DAT, (**e**) 10 DAT, and (**f**) 15 DAT. Scale bars represent 10 μm.

**Figure 2:**
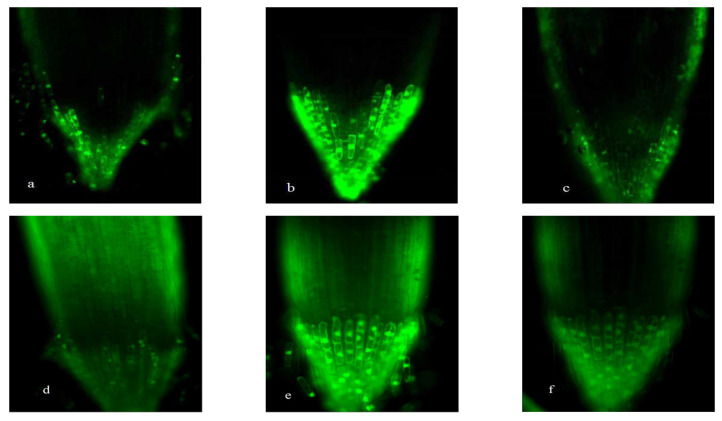
Hydrogen peroxide (H_2_O_2_) distribution in the root tips of HG35 wheat seedlings grown on a medium of two Pi concentrations and sampled at different ages. The treatment (+P): (**a**) 5 DAT, (**b**) 10 DAT, and (**c**) 15 DAT. The treatment (−P): (**d**) 5 DAT, (**e**) 10 DAT, and (**f**) 15 DAT. Scale bars represent 10 μm.

**Figure 3:**
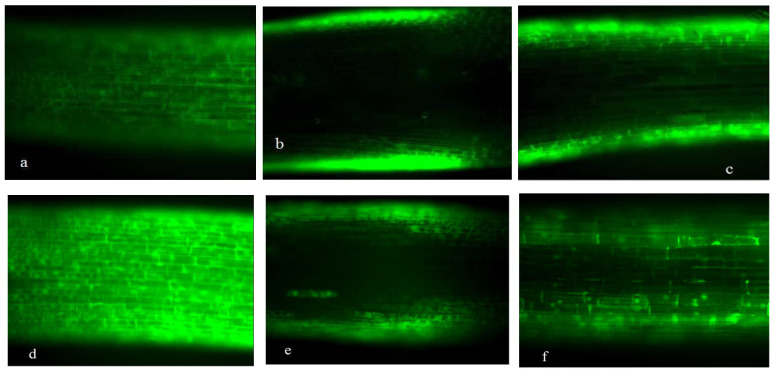
Hydrogen peroxide (H_2_O_2_) distribution in the elongation zone of roots of SM15 wheat seedlings on a medium of two Pi concentrations and sampled at different ages. The treatment (+P): (**a**) 5 DAT, (**b**) 10 DAT, and (**c**) 15 DAT. The treatment (−P): (**d**) 5 DAT, (**e**) 10 DAT, and (**f**) 15 DAT. Scale bars represent 10 μm.

**Figure 4:**
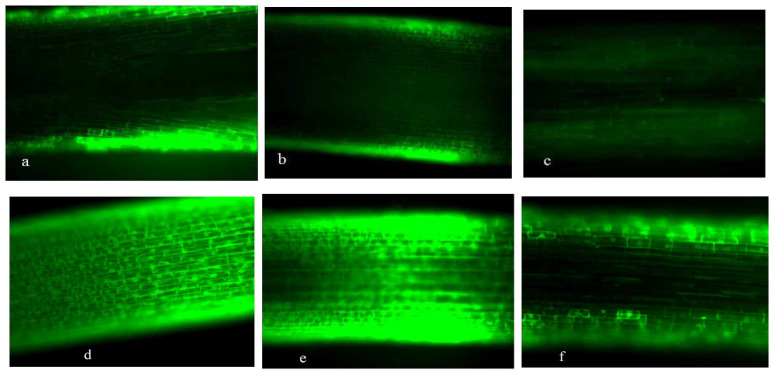
Hydrogen peroxide (H_2_O_2_) distribution in the elongation zone of roots of HG35 wheat seedlings grown on a medium with two Pi concentrations and sampled at different ages. The treatment (+P): (**a**) 5 DAT, (**b**) 10 DAT, and (**c**) 15 DAT. The treatment (−P): (**d**) 5 DAT, (**e**) 10 DAT, and (**f**) 15 DAT. Scale bars represent 10 μm.

Figure 1, Figure 2, Figure 3 and Figure 4 revealed progressive H_2_O_2_ accumulation in root tips of wheat seedlings under both low-P and normal-P conditions, with fluorescence intensity positively correlating with culture duration. P deficiency induced pronounced oxidative responses: root caps and elongation zones exhibited stronger fluorescence under low-P versus normal-P conditions at equivalent growth stages, confirming P deprivation as a potent ROS inducer (Figure 1, Figure 2, Figure 3 and Figure 4).

Under high-P conditions, H_2_O_2_ predominantly localized to the epidermis and proximal regions of root apices, with minimal detection in internal tissues. Conversely, low-P-grown plants exhibited intracellular H_2_O_2_ enrichment in root apices. Both SM15 and HG35 varieties showed stress-responsive spatiotemporal patterns: root tips displayed negligible fluorescence at 5 DAT under P sufficiency (Figure 1a and Figure 2a), whereas low-P induced progressive H_2_O_2_ accumulation peaking in root cap-elongation zone complexes (Figure 1d and Figure 2d). Elongation zone staining dynamics mirrored root tip responses, revealing apoplastic oxidative bursts concentrated in cell wall compartments under P deprivation (Figure 3 and Figure 4). Notably, fluorescence intensity in elongation zones declined from 5 to 15 DAT under sustained low-P stress, suggesting temporal modulation of ROS homeostasis. In particular, staining was most intense in the cell wall compartment of the elongation zone (Figure 3 and Figure 4d–f). Plants grown under P-deficient conditions were characterized by increased levels of ROS in the cell walls when compared to those cultured under high P conditions (Figure 3 and Figure 4). This spatiotemporal patterning—early-phase oxidative burst (5 DAT) transitioning to metabolic quenching—suggests compartmentalized ROS signaling as an adaptive strategy for P acquisition.

### 2.5. SOD, POD, and CAT Activities in the Roots

**Figure 5:**
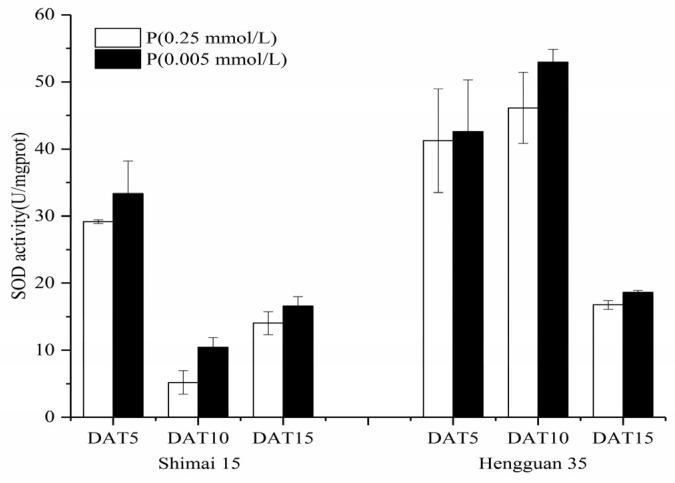
Superoxide dismutase (SOD) activity of wheat seedlings in the different Pi treatments at different root ages. Data represent mean values (±SD) of three independent experiments.

The wheat varieties SM15 and HG35 exhibited P regime-dependent antioxidant enzyme specialization, with SOD activity under low-P showing higher than P-sufficient conditions at critical developmental stages (days 10–15; *p* < 0.05; Figure 5). Temporal dynamics revealed divergent SOD oscillation patterns: SM15 displayed high-low-high activity phases coinciding with fluorescence intensity fluctuations, while HG35 followed inverse low-high-low cycles under P deprivation (Figure 1 and Figure 2). These age- and genotype-specific enzymatic rhythms mirrored root ROS accumulation profiles, suggesting coordinated antioxidant modulation during P-stress adaptation.

**Figure 6:**
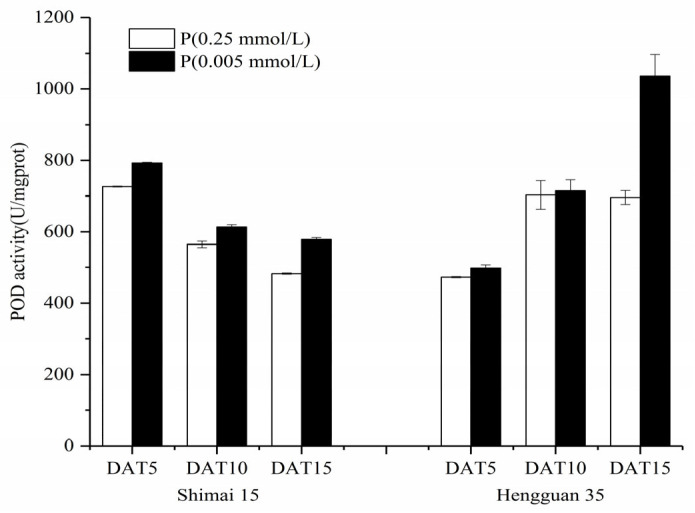
Peroxidase (POD) activity of the different Pi treatments in different aged roots. Data represent the mean values (±SD) of three independent experiments.

An increase in total POD activity was detected under P-deficient conditions (Figure 6) compared to that in roots growing under sufficient-P conditions. As culture time was extended, the POD activity of the 0.005 mmol/L P treatment and the 0.25 mmol/L P treatment tended to decline in SM15, whereas it intensified in HG35 (Figure 6).

**Figure 7:**
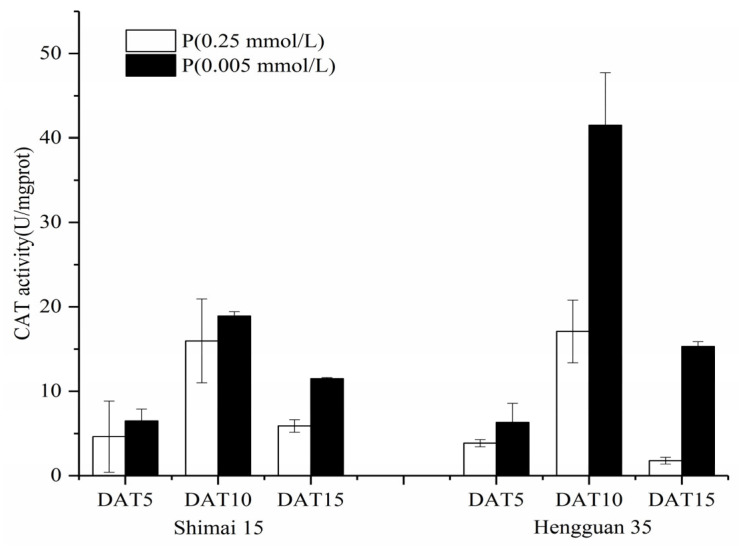
Catalase (CAT) activity in the different Pi treatments of different aged roots. Data represent the mean values (±SD) of three independent experiments.

CAT exhibited biphasic low-high-low rhythms across P regimens, peaking at 10 DAT in SM15 (2.9~3.5-fold increase vs. 5 DAT) and in HG35 (4.4~6.7-fold increase vs. 5 DAT; *p* < 0.01). These oscillations inversely correlated with earlier H_2_O_2_ accumulation patterns (Figure 1, Figure 2, Figure 3 and Figure 4), suggesting compensatory antioxidant activation during prolonged oxidative stress (Figure 7).

## 3. Discussion

### 3.1. P Deficiency Affects the Root Growth of Wheat Seedlings

P utilization efficiency can be enhanced through strategic modulation of RSA to optimize P acquisition and remobilization mechanisms. Under P-deficient conditions, plants exhibit adaptive morphological responses, including (1) preferential carbon allocation to root systems, (2) proliferation of lateral roots, and (3) elevated root-to-shoot biomass ratios—evolutionary strategies to maximize rhizosphere exploration and P capture efficiency [16,17,18,19,20]. Our experimental findings align with these established physiological paradigms (Table 1): 6.8~15.4% increase in root biomass, 31.3~35.1% reduction in shoot biomass, significant depression of shoot P concentration, root/shoot ratio escalation from 0.22 to 0.36. This carbon-partitioning shift reflects source–sink reprogramming wherein photoassimilates are preferentially allocated to subterranean organs, a metabolic trade-off favoring soil exploration over aerial biomass accumulation [21,22]. The root/shoot ratio elevation under P deprivation predominantly stems from shoot growth inhibition rather than absolute root biomass gain, a phenomenon extensively documented in wheat [17,21,22].

In strongly P-impoverished environments, RSA traits such as root hair length and root elongation rate become more critical determinants of P uptake than the kinetic properties of phosphate transporters [1]. Our observations that low-P supply increased primary root length (17.8~18.5%) and average lateral root length (14.7~34.4%), while reducing root length density (8.3–11.4%) and lateral root density (25~27.5%) (Table 2) suggest a strategy that favors the extension of axial roots over the production of fine lateral roots (diameter ≤ 0.2 mm). This aligns with findings that under a severe P limitation, wheat may optimize soil exploration by maintaining primary root growth at the expense of lateral root proliferation—a carbon-saving adjustment consistent with the high metabolic cost associated with fine root turnover [23,24].

The observed reduction in lateral root density under P deficiency contrasts with some reports of lateral root promotion in wheat [21,22,25,26], but agrees with others noting inhibition under sustained low-P stress [24]. Such discrepancies likely reflect genotypic differences, variations in experimental P gradients, and stress duration. Indeed, transient early proliferation of lateral roots may occur, but this response often diminishes as stress intensifies [27,28], which could explain the attenuation of lateral root initiation observed in our study after 15 days of treatment.

From an ecological perspective, the RSA adjustments seen here—prioritizing root elongation over lateral branching—resemble the “topsoil foraging” strategy described in annual crops under P limitation. In contrast, species adapted to extremely P-impoverished soils often employ more specialized “P-mining” strategies, such as the release of carboxylates (e.g., citrate) from cluster roots, which are highly effective in mobilizing sparingly available soil P [1,2]. While wheat does not form cluster roots, the observed architectural shifts may similarly enhance P acquisition by increasing root contact with soil volumes where P is relatively more accessible.

In summary, P deficiency reshapes wheat RSA through integrated morphological and physiological adjustments that enhance soil exploration while balancing carbon costs. Breeding for root traits that improve P capture under low-P conditions—such as longer and denser fine roots in topsoil layers—remains a promising avenue for improving P-use efficiency in wheat, particularly when combined with management practices that optimize rhizosphere processes [2].

### 3.2. P Deficiency Affects ROS Distribution in Distal Parts of Wheat Roots

Spatiotemporal mapping of ROS distribution—particularly H_2_O_2_ in root apical microdomains—is critical for deciphering P-deficiency responses in wheat. Using fluorescein-based histochemical localization, we identified distinct H_2_O_2_ accumulation patterns in root tips (cell division, elongation, and differentiation zones) under contrasting P regimes (0.005 vs. 0.25 mmol/L P). The primary root apex has been reported to harbor two areas of ROS production: the quiescent center and the elongation zone [29,30]. The oxidative environment in the quiescent center is important for maintaining the low cell division rate characteristic of this region [10,29]. In our study, the typical pattern of ROS distribution, with the local maxima in the cell wall of the division and elongation zones, was most evident in roots grown with 0.005 mmol/L P (Figure 1, Figure 2, Figure 3 and Figure 4). In contrast, when the plants were grown on a medium with 0.25 mmol/L P, no distinct fluorescence maximum was detected within the elongation zone (Figure 3 and Figure 4). Our data suggest that the increase in the number of cell divisions and the promotion of cell growth in the root elongation zone, which are responsible for the start-up of primary and lateral root growth in P-deficient wheat, were accompanied by an increase in the ROS distribution pattern typical for growing roots, including elevated ROS levels in the elongation zone (Figure 3 and Figure 4).

Our observations align with reports in *Arabidopsis*, where P deficiency restricts the fluorescence staining maximum to the elongation zone as lateral root primordia develop [9,11]. ROS production in the elongation zone is a common feature of seed plants and is linked to increased cell wall extensibility through mechanisms such as an elevated pectin concentration and cell wall protein activity [31,32,33,34]. While deficient-P conditions can lead to the overaccumulation of ROS and increased callose deposition [12], ROS also participate in cell wall loosening and thereby promote root elongation [12,35]. In wheat, the spatial patterning of H_2_O_2_ appears to integrate developmental reprogramming with cell wall remodeling under P stress.

### 3.3. P Deficiency Affects SOD, POD, and CAT Activity in Roots of Wheat Seedlings

Antioxidant dynamics critically modulate root developmental plasticity under P deprivation through redox-regulated cell proliferation and elongation [36]. Our integrative analysis reveals that sustained P deficiency (15 DAT) induces compartment-specific oxidative reprogramming in wheat roots, characterized by (1) apoplastic H_2_O_2_ maxima in root cap-elongation zone complexes (Figure 1, Figure 2, Figure 3 and Figure 4), (2) 19.8~167.1% elevation in POD activity (*p* < 0.05), and (3) 95.1~759% CAT activation (*p* < 0.01), while constitutive SOD levels were maintained (Figure 5, Figure 6 and Figure 7). The sufficient basal SOD activity enabled the conversion of superoxide (O_2_·^−^) to H_2_O_2_ without a significant increase in total SOD. This is consistent with earlier observations that P-deficient roots exhibit membrane leakiness [37] and increased oxidative stress, as reflected by elevated lipid-peroxidation products (MDA) and cellular H_2_O_2_ [14,38].

The connection between the formation of ROS and enzyme scavenging pathways is well-established. For instance, the overexpression of glutathione reductase (GR) enlarges the ascorbate and glutathione pools, improving root architecture under P stress [39]. In *Arabidopsis*, a set of peroxidases (PODs) targeted by the transcription factor UPB1 controls the superoxide/H_2_O_2_ balance and thereby regulates the transition from cell proliferation to differentiation in the root [40]. Overexpressing of UPB1-targeted POD lengthens the root meristem [40,41]. In our study, the P-deficiency-induced rise in POD and CAT activities coincided with a progressive decline in H_2_O_2_ fluorescence in low-P-treated roots over time (Figure 3, Figure 6 and Figure 7), suggesting a tightly regulated scavenging response. This dynamic aligns with reports of transient H_2_O_2_ bursts coupled with sustained high peroxidase activity under Pi stress [12,42]. Notably, the cultivar HG35, which exhibited greater lateral root length and density than SM15 under stress (Table 2), also showed a significantly stronger induction of POD activity by day 15 (Figure 6). This correlation hints at a potential role for specific POD isoforms in modulating root architecture. In *Arabidopsis*, a set of peroxidases targeted by the transcription factor UPB1 controls the ROS balance between the meristem and elongation zone, thereby regulating the transition from cell proliferation to differentiation, which directly influences root meristem size and root growth [40]. Interestingly, deficiency in the NADPH oxidases AtrbohD and AtrbohF leads to increased POD activity in the mature root zone, contributing to altered O_2_·^−^ accumulation and elevated lateral root density [43]. This interplay between NADPH oxidase-derived ROS and peroxidase activity provides a potential mechanistic framework for interpreting our observations in wheat.

### 3.4. Concentration-Dependent Roles of H_2_O_2_ in Modulating Wheat Root Architecture Under Low P

H_2_O_2_ is a well-documented regulator of root development, promoting nodal root formation in mung bean and cucumber explants [44,45], modulating lateral root growth in legumes and *Arabidopsis* [9,34,46], and altering cell wall composition in root elongation zones [40,47]. In wheat, we observed concentration-dependent dual effects: 10 mmol/L H_2_O_2_ suppressed the total root length (70.6~76.9% reduction) and lateral root length (79.9~84.6% decrease) while stimulating the formation of the nodal root; conversely, 1 mmol/L treatment increased lateral root density by 30.1~65.8% (Table 2). This biphasic response differs from that in *Arabidopsis*, where ≤10 µmol/L H_2_O_2_ had little effect on root length, and higher concentrations inhibited elongation [48], highlighting species- and concentration-specific sensitivities.

The stimulation of lateral root density by 1 mmol/L H_2_O_2_ was more pronounced under P-deficient conditions, suggesting cross-talk between ROS signaling and P-starvation pathways. Mechanistically, ROS are known to promote cell wall loosening, a process critical for both cell elongation and the emergence of lateral root primordia through overlying tissues. Our observation that P starvation triggered ROS accumulation in the elongation zone (Figure 3 and Figure 4), correlating with enhanced root elongation, supports this view. Exogenous ·OH promotes maize root elongation via wall extension, and loose walls themselves enhance ·OH production in the elongation zone—effects that are counteracted by ROS inhibitors [30]. The regulatory network is precise; for instance, in *Arabidopsis*, the transcription factor UPB1 represses specific peroxidases in the elongation zone to maintain the low H_2_O_2_ levels necessary for meristem activity. Disrupting this balance (e.g., in upb1 mutants or UPB1-overexpressors) alters the meristem size and H_2_O_2_ distribution [40,47]. While our study did not localize H_2_O_2_ specifically in lateral root primordia, the enhanced fluorescence in the elongation zone and cell walls under low P is consistent with models where localized ROS facilitate cell wall remodeling for lateral root development [48,49]. The inhibitory effect of 10 mmol/L H_2_O_2_ on lateral roots may result from a supra-optimal ROS level that disrupts meristematic activity or triggers different signaling cascades, possibly analogous to the MYB30-mediated suppression of cell cycle genes by high H_2_O_2_ [50].

The induction of nodal roots by 10 mmol/L H_2_O_2_ (Table 2) aligns with previous reports in other species [44,45]. The antioxidant AsA also increased nodal root numbers, and co-application with 1 mmol/L H_2_O_2_ had a synergistic effect. However, AsA eliminated the inductive effect of 10 mmol/L H_2_O_2_ in some conditions (Table 2). This highlights the complex redox interplay in vivo. It is important to clarify that AsA does not directly scavenge H_2_O_2_; rather, it serves as the specific electron donor for ascorbate peroxidase (APX) in the Halliwell–Asada cycle. Furthermore, AsA can influence root development by modulating peroxidase-mediated processes like lignification, as it scavenges phenoxy radicals generated during cell wall stiffening reactions. The effects of DPI (an inhibitor of NADPH oxidases like RboH) further implicate enzymatic ROS production in root morphogenesis. DPI suppression of root growth was partially rescued by co-application with H_2_O_2_, indicating that exogenous H_2_O_2_ can compensate for the loss of RboH-derived ROS in certain pathways (Table 2). This is consistent with findings where DPI inhibited peroxidase-mediated ROS production and lateral root formation, effects reversible by H_2_O_2_ addition [7,46].

Regarding nodal roots, 10.0 mmol/L H_2_O_2_ significantly promoted their formation, whereas 1 mmol/L H_2_O_2_ had no effect (Table 2)—consistent with earlier reports in mung bean and cucumber explants [44,45]. AsA alone increased nodal root numbers in SM15 across P treatments and in HG35 under sufficient P; co-application with 1 mmol/L H_2_O_2_ produced a synergistic promotion. However, AsA eliminated the inductive effect of 10 mmol/L H_2_O_2_ on nodal roots in SM15 under sufficient P, contrasting with the transient stimulation seen in mung bean [45]. DPI strongly suppressed nodal root formation, and H_2_O_2_ co-treatment reversed this inhibition, confirming that DPI acts primarily by blocking cellular H_2_O_2_ production [44,45]. Collectively, these results underscore the dose-dependent, context-specific roles of H_2_O_2_ in shaping wheat RSA under varying P availability.

### 3.5. Potential Interactions with Other Reactive Molecular Species

Under P-deficient conditions, a sophisticated signaling network involving ROS, reactive nitrogen species (RNS), and reactive sulfur species (RSS) is activated to reshape RSA and enhance P acquisition. ROS, particularly H_2_O_2_, act as early signals that influence lateral root development and root hair elongation, integrating nutrient status with developmental programs [49,51]. Concurrently, nitric oxide (NO), a key RNS, plays a central role in mediating root responses to P stress. It fine-tunes root growth by enhancing P remobilization and synergizing with hormonal pathways, including auxin and strigolactones, to promote root elongation under combined nutrient limitations [52,53,54]. Similarly, hydrogen sulfide (H_2_S) and related RSS have emerged as crucial regulators. H_2_S enhances lateral root formation, improves antioxidant capacity, and contributes to ion homeostasis, thereby supporting root adaptation to P deficiency and other concurrent abiotic stresses [52,55].

Critically, these reactive molecules do not function in isolation but engage in extensive cross-talk. Their interactions often occur at the level of protein post-translational modifications. For instance, NO-mediated S-nitrosylation and H_2_S-induced persulfidation can target the same cysteine residues on regulatory proteins, creating a dynamic, competitive interplay that finely modulates protein function and downstream signaling [55]. The tripeptide GSH serves as a major metabolic and redox hub connecting these pathways. NO can react with GSH to form S-nitrosoglutathione (GSNO), a bioactive NO reservoir, while H_2_S metabolism interacts with the GSH pool, affecting cellular redox buffering and the ascorbate-glutathione cycle critical for stress tolerance [51,56]. Furthermore, ROS, RNS, and RSS signals are integrated with phytohormone signaling networks to coordinately regulate cell division, elongation, and differentiation in the root, ultimately optimizing RSA for improved P foraging [51]. Future research employing multi-omics approaches is needed to map the spatiotemporal dynamics and precise protein targets of this interactive redox network, which holds promise for developing crops with enhanced nutrient-use efficiency.

## 4. Materials and Methods

### 4.1. Plant Materials and Growing Environment

Two varieties of wheat, SM15 and HG35, were used. They are drought-resistant and high-yield varieties widely cultivated in the North China Plain.

The seed surface was disinfected for 30 min with 1% (*v*/*v*) NaClO, rinsed in deionized water, and placed in Petri dishes to germinate for 2 days in the dark at 25 °C and a humidity of 70%. The germinated seeds were transferred into quartz sand to grow for 7 days, consistently growing seedlings at two-leaf stage were selected to transplant to a glass tube (5 cm diameter and 20 cm high) filled with 0.5 L of nutrient solution. The concentrations of nutrient solutions were as follows (in mmol/L): K_2_SO_4_ 0.5, KCl 0.35, Ca(NO_3_)_2_ 2.0, MgSO_4_·7H_2_O 0.6, EDTA-Fe(Ⅱ) 4.0 × 10^−2^, H_3_BO_3_ 1.0 × 10^−3^, MnSO_4_·H_2_O 1.0 × 10^−3^, ZnSO_4_·7H_2_O1.0 × 10^−3^, CuSO_4_·5H_2_O 1.0 × 10^−4^, and (NH_4_)Mo_7_O_24_·4H_2_O 5.0 × 10^−6^. The pH was adjusted to 6.0 with 0.1 mol/L NaOH or 0.1 mol/L HCl. The nutrient solution was continuously aerated and changed every 48 h to minimize nutrient depletion. The containers were parceled with black plastic. The plants were grown in a growth chamber, in which light intensity was set at approximately 500~600 μmol m^−2^∙s^−1^ with 14h per day and the temperature was maintained at 26 °C/22 °C day/night.

### 4.2. Treatments

This experiment employed the following: two P concentrations (low P (0.005 mmol/L) and optimal P (0.25 mmol/L)); two H_2_O_2_ concentrations (low concentration (1.0 mmol/L) and high concentration (10.0 mmol/L)); and AsA and DPI applied at 2.5 mmol/L and 5.0 µmol/L [10,45,48], respectively. The treatment combinations were as follows: (1) P (0.005 or 0.25 mmol/L) + H_2_O_2_ (0.0, 1.0, or 10.0 mmol/L); (2) P (0.005 or 0.25 mmol/L) + AsA (2.5 mmol/L); (3) P (0.005 or 0.25 mmol/L) + DPI (5.0 µmol/L); (4) P (0.005 or 0.25 mmol/L) + H_2_O_2_ (1.0 or 10.0 mmol/L) + AsA (2.5 mmol/L); (5) P (0.005 or 0.25 mmol/L) + H_2_O_2_ (1.0 or 10.0 mmol/L) + DPI (5.0 µmol/L). P was supplied in the form of KH_2_PO_4_, and the K^+^ concentration difference was regulated by the supply of KCl in the medium for the low Pi treatment.

After growing in a normal nutrient solution for one week, plants were transplanted and then subjected to Pi treatments. H_2_O_2_ (0, 1, and 10 mmol/L), DPI (0 and 5 μmol/L), and AsA (0 and 2.5 mmol/L) were added to nutrient solution, respectively. Four replicates each treatment were performed. DPI was bought from Toronto Research Chemicals, Toronto, ON, Canada.

### 4.3. Determination of Biomass and Phosphorus Content

The biomass, P content, and root morphology of the wheat seedlings were determined after 15 DAT.

The samples were placed in bags after being washed with deionized water and dried at 105 °C for 30 min; shoot biomass and root biomass were recorded by drying to constant weight at 70 °C. The plant samples were digested with H_2_SO_4_ and H_2_O_2_ and P concentrations in the digest solutions were determined using vanadium molybdate yellow colorimetric method [57].

### 4.4. Determination of Root Development

The root samples were fixed in FAA solution with 38% formaldehyde 5 mL: acetic acid 5 mL: 70% alcohol 90 mL. Roots were placed on a glass plate and separated with water, and the length was measured with a ruler. The number of roots including primary roots, nodal roots and lateral roots was counted.

Digital images of root samples spread with minimum overlaps on an A4-size tray were obtained with a root scanner (EPSON Perfection V750). WinRHIZO (Regent Instruments Inc., Quebec, QC, Canada) software was used to analyze the root images to determine the total root length. Root length density was computed by dividing total root length with root biomass. The total length of lateral root was calculated with total root length minus primary root length and nodal root length. The average length of lateral root was estimated by lateral root length divided by the number of lateral roots. The number of lateral roots on the primary roots and nodal roots per unit length was expressed as lateral root density (no./cm).

### 4.5. Histochemical Localization of H_2_O_2_ with Fluorescein Staining

The ROS distributions were determined in the fresh root samples harvested at 5, 10, and 15 DAT, respectively.

Root tip samples were incubated at 25 °C for 15 min in darkness in 50 mmol/L phosphate-buffered solution (pH 6.1) containing 5 µmol/L dihydrofluorescein diacetate (H_2_FDA; Molecular Probes, Eugene, OR, USA), a fluorescent probe, to detect H_2_O_2_ [58]. After that the samples were rinsed with the phosphate-buffered solution and imaged in a microscope (IBE2003, Chongqing Optoelectronic Instrument Co., Ltd., Chongqing, China). The root tips were mounted on a microscopic slide in a drop of buffer and covered with a coverslip to prevent squashing the root. A 450 to 490 nm excitation filter and a 515 nm emission filter were used. The images were captured and analyzed using Tsview7 software (Tsview7 Application, Chongqing Optoelectronic Instrument Co., Ltd.).

### 4.6. Determination of Activities of SOD, POD and CAT

Fresh roots were sampled at 5, 10, and 15 DAT, respectively. A total of 0.3 g of root samples were placed into centrifuge tubes and frozen immediately in liquid nitrogen for 2 min, after which the sample was ground using a Mixer Mill MM 400 (Retsch GmbH, Haan, Germany). A total of 2 mL of 50 mmol/L pH 7.0 phosphate buffer solution containing 0.1% (*v*/*v*) PVP and 0.1% (*v*/*v*) mercaptoethanol was added after the samples were ground completely. The suspension was centrifuged at 13,000× *g* for 20 min at 4 °C. The supernatant was used for the determination of the activities of SOD, POD, and CAT.

SOD activity was determined using nitro blue tetrazolium (NBT) reduction based on the method of Beauchamp and Fridovich [59]. One unit of SOD (U) was defined as the amount of tissue extract that caused 50% inhibition of the photoreduction of NBT. The corresponding reagents were added to two blanks. One blank tube was covered completely with black paper, and the other tubes were placed in a light incubator for 20–30 min at 25 °C with 600 μmol m^−2^∙s^−1^ light intensity. Absorbance was determined at 560 nm after the reaction. Total POD activity was estimated according to the modified method of Chance and Maehly [60]. The guaiacol was used as an electron donor by measuring its oxidation in a reaction medium containing 2.9 mL of phosphate buffer, 0.5 mL of 2% H_2_O_2_, 0.1 mL of guaiacol, and 0.1 mL of enzyme extracts. The changes in absorbance at 470 nm were recorded per min. One unit of POD (U) was defined according to the change in absorbance. CAT activity was determined in the extracts by measuring the decline in absorbance at 240 nm for 2 min. The reaction medium contained 0.1 mL of 2% H_2_O_2_, 2 mL of phosphate buffer, and 0.1 mL of enzyme extract. The activities of POD and CAT were expressed as the changes in absorbances (ΔA) in one minute per mg protein. The protein content in the supernatant was estimated according to Bradford [61], using bovine serum albumin as a standard. All spectrophotometic analyses were conducted in a UV–VIS spectrophotometer (UV-2700, SHIMADZU Co., Ltd. Kyoto, Japan).

### 4.7. Statistical Analysis

Data expressed as means ± standard deviation (SD) were shown in the figures and tables. Analysis of variance was used to assess the differences among the treatments. A *p*-value at the 5% probability level (*p* < 0.05) was considered significant.

## 5. Conclusions

Under low P availability, wheat roots exhibited elongation of primary and lateral roots but reduced lateral root density. P starvation elevated ROS in the root elongation zone and quiescent center of the primary root and mature lateral root. Critically, ROS accumulation in the elongation zone preceded root elongation initiation in low-P medium, correlating with enhanced cell elongation activity. Oxidative enzyme activity significantly increased under P-starvation versus P-sufficient conditions: POD and CAT activities after 15 DAT, and SOD activity, showed no significant change. Exogenous H_2_O_2_ induced concentration-dependent morphological changes modulated by P availability, and 10 mmol/L H_2_O_2_ treatment increased the number of nodal roots and a reduction in lateral root density. A 1 mmol/L H_2_O_2_ concentration treatment enhanced lateral root density, such that the stimulatory effect was more significant in P-starved wheat.

## Figures and Tables

**Table 1:** Shoot biomass, root biomass, root/shoot ratio and P concentration in shoots of wheat seedlings as influenced by exogenous AsA and DPI under different concentrations of Pi and H_2_O_2_ addition.

Treatments				Shoot Biomass (g/Plant)		Root Biomass (g/Plant)		Root/Shoot Ratio		P Concentration in Shoots (mg/g)	
P (mmol/L)	H_2_O_2_ (mmol/L)	AsA (mmol/L)	DPI (μmol/L)	SM15	HG35	SM15	HG35	SM15	HG35	SM15	HG35
0.005	0	0	0	0.132	0.131	0.045	0.047	0.36	0.36	232.45	231.27
		2.5	0	0.102	0.101	0.022	0.028	0.22	0.27	234.58	225.01
		0	5	0.07	0.091	0.017	0.023	0.24	0.26	269.62	216.67
	1	0	0	0.093	0.106	0.037	0.041	0.41	0.39	256.18	190.63
		2.5	0	0.084	0.102	0.023	0.032	0.28	0.32	241.95	226.18
		0	5	0.08	0.083	0.028	0.028	0.35	0.34	216.99	200.38
	10	0	0	0.082	0.093	0.018	0.026	0.22	0.28	276.01	243.89
		2.5	0	0.076	0.075	0.051	0.057	0.68	0.76	257.73	280.82
		0	5	0.083	0.088	0.022	0.024	0.27	0.28	220.38	182.42
0.25	0	0	0	0.192	0.202	0.039	0.044	0.21	0.22	962.99	916.1
		2.5	0	0.12	0.105	0.029	0.027	0.24	0.27	437.72	487.54
		0	5	0.074	0.089	0.017	0.022	0.23	0.24	462.84	439.78
	1	0	0	0.146	0.138	0.039	0.043	0.27	0.31	806.75	786.86
		2.5	0	0.108	0.12	0.026	0.029	0.24	0.25	497.59	487.52
		0	5	0.107	0.094	0.028	0.028	0.26	0.3	323.35	352.38
	10	0	0	0.084	0.096	0.023	0.022	0.27	0.23	616.52	523.95
		2.5	0	0.09	0.087	0.056	0.058	0.63	0.67	597.07	574.69
		0	5	0.095	0.093	0.026	0.025	0.27	0.27	324.2	334.54
*LSD* (*p* < 0.05)				0.025	0.023	0.006	0.006	0.07	0.05	65.56	64.00

**Table 2:** Total number of nodal roots, total root length, root length density, primary root length, lateral root length, lateral root density and average lateral root length of wheat seedlings as influenced by exogenous AsA and DPI under different concentrations of Pi and H_2_O_2_ addition.

Treatments				Number of Nodal Roots (NO.)		Total Root Length (cm)		Root Length Density (cm/g)		Primary Root Length (cm)		Lateral Root Length (cm)		Lateral Root Density (NO./cm)		Average Lateral Root Length (cm)	
P (mmol/L)	H_2_O_2_ (mmol/L)	AsA (mmol/L)	DPI (μmol/L)	SM15	HG35	SM15	HG35	SM15	HG35	SM15	HG35	SM15	HG35	SM15	HG35	SM15	HG35
0.005	0	0	0	6	5	918.6	903.7	20,424.7	19,151.7	31.1	29.4	706.1	719.6	0.79	1.02	4.3	3.9
		2.5	0	8	6	254.9	249.5	13,319.1	11,846.7	21.7	18.6	131.5	144.8	0.63	0.86	1.7	1.7
		0	5	3	2	229.7	275.3	11,443.4	9049.5	18.6	19.4	127.8	172.8	0.74	0.82	1.7	2.1
	1	0	0	5	4	338.7	348.8	9138.0	8466.5	25.2	25.6	184.9	206	1.31	1.53	0.9	1
		2.5	0	9	6	238.4	281.1	9141.5	9102.8	19.7	20	127.4	173.3	0.54	0.69	2.1	2.4
		0	5	8	5	256.7	256.6	10,354.1	8830.6	20.8	19.6	137.3	151.6	1.1	1.38	1	1
	10	0	0	10	10	221.0	252.4	12,600.3	9906.7	19.7	20.5	108.4	134.2	0.87	1.05	1.1	1.1
		2.5	0	9	8	295.2	305.3	11,393.5	10,071.7	20.5	19.5	171.8	194.6	0.58	0.68	2.4	2.6
		0	5	11	10	253.6	243.1	5751.1	5351.1	20.1	17.6	136.4	140.4	1.1	1.22	1	1.1
0.25	0	0	0	7	6	875.5	941.2	22,272.7	21,620.1	26.4	24.8	681.2	770.8	1.09	1.36	3.2	3.4
		2.5	0	10	7	287.1	249.5	11,563.5	12,123.1	20.8	17	149.3	150.2	0.65	0.76	1.7	1.9
		0	5	3	2	191.9	263.7	9829.9	9116.7	17.1	18.1	93.9	168	0.54	0.83	1.8	2.1
	1	0	0	7	5	404.2	411.2	10,246.7	9682.7	23.3	25.3	232.6	255	1.58	1.77	0.9	0.9
		2.5	0	10	8	294	261.8	9157.0	8975.7	20.2	15.1	166.8	165	0.57	0.71	2.4	2.4
		0	5	11	8	259.2	252	11,664.9	8945.5	19.5	19.2	137.7	150.7	0.96	1.24	1.2	1.2
	10	0	0	16	11	257.5	217	11,434.8	10,082.5	19.2	18	136.8	124.8	0.83	1.06	1.4	1.3
		2.5	0	12	10	336.3	301.4	10,698.9	10,499.5	20.5	17.3	193.5	188.2	0.50	0.59	2.7	2.9
		0	5	14	10	268.4	258.4	6122.0	5249.3	21.2	17.8	140.5	154	1.12	1.38	1	1.1
*LSD* (*p* < 0.05)				2	1	62.6	61.7	1477.9	1034.2	2.3	2.1	54.4	52.7	0.1	0.1	0.4	0.4

## Data Availability

All data generated or analyzed during this study are included in this published article.
